# Specific and sensitive detection of the conifer pathogen *Gremmeniella abietina *by nested PCR

**DOI:** 10.1186/1471-2180-5-65

**Published:** 2005-11-09

**Authors:** Qing-Yin Zeng, Per Hansson, Xiao-Ru Wang

**Affiliations:** 1 National Institute for Working Life, SE-90713 Umeå, Sweden; 2 Department of Molecular Biology, Umeå University, SE-90187 Umeå, Sweden; 3 Department of Silviculture, the Swedish University of Agricultural Sciences, SE-90183 Umeå, Sweden

## Abstract

**Background:**

*Gremmeniella abietina *(Lagerb.) Morelet is an ascomycete fungus that causes stem canker and shoot dieback in many conifer species. The fungus is widespread and causes severe damage to forest plantations in Europe, North America and Asia. To facilitate early diagnosis and improve measures to control the spread of the disease, rapid, specific and sensitive detection methods for *G. abietina *in conifer hosts are needed.

**Results:**

We designed two pairs of specific primers for *G. abietina *based on the 18S rDNA sequence variation pattern. These primers were validated against a wide range of fungi and 14 potential conifer hosts. Based on these specific primers, two nested PCR systems were developed. The first system employed universal fungal primers to enrich the fungal DNA targets in the first round, followed by a second round selective amplification of the pathogen. The other system employed *G. abietina*-specific primers in both PCR steps. Both approaches can detect the presence of *G. abietina *in composite samples with high sensitivity, as little as 7.5 fg *G. abietina *DNA in the host genomic background.

**Conclusion:**

The methods described here are rapid and can be applied directly to a wide range of conifer species, without the need for fungal isolation and cultivation. Therefore, it represents a promising alternative to disease inspection in forest nurseries, plantations and quarantine control facilities.

## Background

*Gremmeniella abietina *(Lagerb.) Morelet is among the most destructive conifer forest pathogens in the northern hemisphere. This ascomycete fungus has a broad host range and causes stem canker and shoot dieback in many conifer species of the genera *Pinus*, *Abies*, *Picea*, *Larix*, *Tsuga *and *Pseudotsuga *[1-3]. In Sweden, *G. abietina *is found on the two native conifers *Picea abies *and *Pinus sylvestris*, as well as the introduced species, *Pinus contorta *[4-6].

Under favorable conditions, the life cycle of *G. abietina *takes two years to complete [5]. The fungus may grow in the host as an endophyte for more than one year [7,8], and infected trees can remain undetected for several years before manifesting visible symptoms. This poses difficulties for diagnosing disease at an early stage, using asymptomatic materials. Nursery seedling inspection and quarantine control require sensitive detection methods to limit the spread of the pathogen. Various morphological, physiological, pathogenic and biochemical characters and molecular markers have been employed to distinguish and characterize different races and types of the fungus, such as the North American race (NA), European (EU) race, and large tree type (LTT) and small tree type (STT) [9-15]. Most of these methods require isolation of the fungus in culture. This process is time consuming and is not appropriate for the detection of the fungus directly in infected but asymptomatic tissues.

Specific polymerase chain reaction (PCR) based detection methods are sensitive and robust techniques when used in plant disease diagnostic research. By employing specific PCR primers it is possible to selectively amplify the pathogen from infected tissues without the need for isolation. Among the different PCR techniques, nested PCR (a two-step PCR system in which the first round PCR products are subjected to a second round PCR amplification with more specific primers) is extremely sensitive and allows the detection of a fungal pathogen in minute amounts of infected material [16-18]. Recently, a nested PCR procedure was developed for the detection of *G. abietina *[19]. This method is based on the polymorphic sites in the ribosomal DNA (rDNA) internal transcribed spacer (ITS). The ITS evolves rapidly and significant variations within a species or even within a genome have been reported for fungi and plants [20-23]. The specific markers from the ITS region are, therefore, potentially unstable because of the high mutation rate, and would need to be validated by extensive sample testing. The 18S rRNA gene is much more conservative compared to the ITS. Specific markers developed from this DNA region are less likely to be invalid due to intraspecific variations producing false negative detections.

The aim of the present study was to develop a stable, specific and sensitive method for detecting *G. abietina *infection in a broad range of hosts. There was no intention to differentiate between the races/types of the fungus, which would be difficult using this conserved DNA segment. The 18S rRNA gene was sequenced from *G. abietina *isolated from four host species. Highly specific primers were designed for *G. abietina*, based on extensive sequence homology analysis. Two nested PCR systems were developed for sensitive detection of the pathogen in host tissues. The first system employed fungal universal primers in the first round PCR, to enrich the fungal rDNA in the plant genomic background, followed by the specific amplification of *G. abietina *rDNA in the second round PCR. The second system employed *G. abietina *specific primers in both PCR rounds. Both approaches can detect the presence of *G. abietina *in composite samples with high sensitivity. This procedure is rapid and can be used directly on plant materials without the need for fungal isolation and subsequent cultivation. The methods described here represent a promising alternative to disease inspection in forest nurseries, plantations and quarantine control facilities.

## Results

### Specific amplification of *G. abietina*

The fungal universal primer pair NS1/8 gave an amplification product of about 1700 bp for all of the 58 fungal strains tested, except *Stachybotrys bisbyi *which produced a larger PCR fragment (Fig. 2A). None of the 14 conifer species produced any amplicon with this primer pair, demonstrating the incompatibility of the primers to conifer 18S rDNA. The two pairs of specific primers, NS.Grem3/4 and NS.Grem5/6, amplified fragments of 1080 bp and 837 bp, respectively, only in *G. abietina *(Fig. 2B, C). No amplified products were generated from samples of any of the other fungi and plants with these two primer pairs. When used as inner primers in nested PCR, in combination with NS1/8 as outer primers, NS.Grem3/4 and NS.Grem5/6 gave amplification patterns identical to those in corresponding single-step PCR assays. The nested PCR using NS.Grem3/4 as outer primers and NS.Grem5/6 as inner primers gave an amplification pattern identical to that of NS.Grem5/6. No amplifications were observed for any of the plants and fungi except for *G. abietina *by any of the nested PCR (amplification pattern identical to Fig. 2B, C, thus not shown). These tests were performed in 3 – 5 replicates and the same specific amplification pattern was observed. The only difference between the nested PCR and the single-step specific PCR was that the amplification signals were stronger for the nested PCR. Thus, the prime pairs NS.Grem3/4 and NS.Grem5/6 are highly specific to *G. abietina *at annealing temperatures of 60°C and 56°C, respectively, both in single-step specific PCR and in two-step nested PCR. The lack of cross-amplification from any of the conifer species in either of the PCR systems demonstrates the scope for specific detection of the pathogen in all these hosts.

### Evaluation of detection sensitivity

If the method is to be used for the early detection of infection in bulked samples in forest practice, a high level of sensitivity is required. Three different tests using a dilution series of *G. abietina *genomic DNA, with and without other background DNA, were conducted to compare the detection limits of the different PCR setups. The lowest DNA dilution that could provide a reproducible, unambiguous visible signal (in 3 μl PCR product) on ethidium bromide stained gels after electrophoresis was defined as the PCR detection limit.

Both the universal primers and the specific primers were tested first in single-step PCR assays. As shown in Fig. 3, all three pairs of primers can detect 0.15 ng DNA template. Among the three primer pairs, NS.Grem3/4 gave the strongest amplification signal indicating greater PCR efficiency than the other two. Nevertheless, the magnitude of detection sensitivity was the same for all three single-step PCR systems. By combining NS1/8 amplification with a second round amplification using either of the specific primer pairs in a nested PCR, the detection sensitivity dramatically increased. As little as 15 fg *G. abietina *DNA can be detected, approximately equivalent to a single fungal genome (Fig. 4a, b Test 1). The nested PCR using NS.Grem3/4 followed by NS.Grem5/6 gave a detection sensitivity similar to the two NS1/8-based methods though with even stronger amplification signal (Fig. 4c Test 1). Thus, the nested PCR is about 10,000 times more sensitive than the single step PCR assays.

To simulate the detection of *G. abietina *in a host's genomic background, the dilution series of *G. abietina *DNA was mixed with *P. contorta *DNA in equal volume and subjected to nested PCR analysis (Test 2, Table 2). The amplification resulted in a detection limit of 7.5 fg *G. abietina *DNA in the background of 6 ng of *P. contorta *DNA (i.e. a millionth of the quantity of host DNA, Fig. 4 Test 2). This test was performed in 3 – 5 replicates and the same magnitude of detection sensitivity was observed. This result is very similar to the detection limit achieved with *G. abietina *DNA alone (Fig. 4 Test 1). Thus, the presence of *P. contorta *DNA, even at very high relative concentrations, did not affect the efficiency of the nested PCR for detecting *G. abietina*.

To simulate the detection of *G. abietina *in a composite fungal background, the dilution series of *G. abietina *DNA was mixed with equal volumes of DNA from seven other fungi (Test 3, Table 2). In contrast to the previous two tests, this composite fungal DNA produced a visible amplification product (ca. 1.7 Kb in size) in all seven DNA dilution mixtures after the first round PCR with universal fungal primers (data not shown). This was due to the presence of background fungal DNA (ca. 9 ng) in all PCR mixes, no matter how little *G. abietina *DNA was present: the NS1/8 primers were compatible with all of the fungi in the mixture. Following the nested PCR, the detection limit was 0.075 ng *G. abietina *DNA in the background of 9 ng of other fungal DNA (i.e. about a hundredth of the fungal background DNA, Fig. 4a, b Test 3). This is a pronounced decrease in detection sensitivity compared to Tests 1 and 2. In contrast, the detection sensitivity of the nested PCR using the specific primers in both PCR steps was not affected by the presence of other fungal DNA, and gave the same detection sensitivity as in Test 1 and 2 (Fig. 4c Test 3).

### Detection of *G. abietina *infection in *Pinus contorta* by nested PCR

To test the ability of the nested PCR systems to detect directly *G. abietina *infection in conifer trees, both brown and green needles from the infected trees of *P. contorta *were used for DNA isolation and PCR amplifications. All the nested PCR assays detected *G. abietina *in the needle samples from the infected twigs, which gave clear products of 1080 bp and 837 bp long with the inner primer pairs NS.Grem3/4 and NS.Grem5/6, respectively (Fig. 5). Neither the healthy *P. contorta *sample, nor the negative control produced any amplification product (Fig. 5). This verifies that the nested PCR product from the needle samples was amplified from *G. abietina*.

## Discussion

*Gremmeniella abietina *is widespread and causes severe damage to several conifer species. Large-scale forest epidemics have been reported from several continents [1,6,24-26]. The disease can spread through infected seedling nurseries. Intercontinental migration of the pathogen has also been reported as a result of international transportation of infected forest materials [27,28]. Rapid detection methods that can be applied directly to asymptomatic tissues would be valuable for forest disease management. Previously reported methods for the morphological, biochemical, pathogenic or genetic characterization of this fungus require its isolation in culture [14,28-31]. Such characterization allows the species to be subdivided into different races and types. However, host differentiation of the fungus is very limited and different races/types can coexist in the same geographic region and infect the same host species [27,29,30,32,33]. Therefore, for forest management, a general detection method for *G. abietina*, regardless of race/type, would be highly desirable. The identical 18S rDNA sequences from *G. abietina *of NA, EU, LTT and STT race/type isolated from different hosts indicate that, in this fungus, the sequence is conservative. Therefore, markers based on it exhibit general intraspecific applicability. A high specificity of the detection system is a prerequisite for its application in pathogen diagnosis. The specific primers developed in this study successfully detected *G. abietina *at species level and, thus, can function as rapid molecular markers for its identification and detection in composite fungal or plant samples without the need for isolation and cultivation. By verifying these DNA markers in a wide range of conifer species, the present study indicates that this detection system can be applied to all potential hosts, so it should be a valuable forest management tool across broad geographic regions.

Apart from the specificity, the sensitivity of a detection system is also important for early infection diagnosis, particularly in bulk samples. The sensitivity of a PCR assay depends on several factors, most importantly on the primer composition, structure and homology to the target molecule. In this study, when the three pairs of primers, NS1/8, NS.Grem3/4 and NS.Grem5/6, were tested on *G. abietina *DNA in single-step PCR with the same number of cycles, NS.Grem3/4 was found to be ca. 10 times more efficient than the other two pairs. The higher amplification efficiency of NS.Grem3/4 was consistent across all PCR assays (Fig. 4). Thus, careful design and selection of the primers can significantly improve the sensitivity of a PCR assay.

Nested PCR was employed in this study to improve the detection sensitivity of the pathogen in the hosts. The use of universal fungal primers in the first round PCR enriches the fungal rDNA in the plant genomic background, then, in the second round, there is selective amplification of the target pathogen. This approach is particularly attractive when screening for multiple fungal pathogens in minor amount of plant tissue. The nested PCR developed in this study can detect as little as a single fungal genome even in high background levels of pine DNA. The template DNA concentration and composition can influence the efficiency of nested PCR. In the presence of a high proportion of other fungal DNA the detection limit of nested PCR with outer primers being the fungal universal primers NS1/8 was significantly decreased, mainly due to primer competition in the first round PCR. Since the universal primers are compatible with all the fungi in the mixture, the very small proportion of *G. abietina *present (<0.1%) would have little chance to compete for the primers. The target template was, therefore, not enriched in the first round PCR, which in turn affected the nested PCR sensitivity. This problem can be avoided by employing *G. abietina*-specific primers in both PCR rounds. The nested PCR based on this approach showed high detection sensitivity for *G. abietina *even in a high background of other fungal DNA. In real situations, the detection systems would usually be used on either suspected *G. abietina *infections or asymptomatic tissues. DNA isolated from these materials would mostly comprise the host DNA and DNA from endophytic fungi. *G. abietina *may or may not be the major component among the endophytic fungi. Thus, the nested PCR system using specific primers in both PCR steps would satisfy both the specificity and sensitivity requirements for diagnostic applications.

In the analysis of plant samples, extraction of sufficient fungal genomic DNA is also important since the fungal tissue is usually present at low levels relative to the amount of host tissue. If this is not achieved, the detection sensitivity may be inadequate and could result in a false negative. For large conifer trees, the stage and degree of the disease development as well as tissue sampling position would also affect the pathogen's detection. In the early stage of infection the amount and the spread of fungal mycelia is limited. Tissues from parts of the tree other than the close vicinity of the infection site may give negative detection. Thus, for large conifers multiple samples should be collected from the suspected tree for examination.

## Conclusion

This study developed rapid, specific and sensitive detection systems for the conifer pathogen *G. abietina*. The specific markers were validated for a broad range of conifer hosts and fungi. Thus, the detection methods described here could have broad applications in forest protection and disease management programs. It should be also recognized that different race/types of *G. abietina *have distinct epidemiological and aetiological attributes and the ideal molecular assay should allow the user to identify not only the species but also the races and biotypes. The assay reported here could be used in combination with other race or biotype-specific assays [12,19], either in a multiplex or in a sequential fashion, to better understand the distribution and disease development of different *G. abietina *infections.

## Methods

### Fungal strains, plant species and genomic DNA extraction

*Gremmeniella abietina *was isolated from *Pinus sylvestris, P. contorta, P. resinosa and P. banksiana *(Table 1). Eleven isolates representing NA, EU, LTT and STT race/type were sequenced for the 18S rRNA gene (Table 1). Thirty-one other fungal strains, from 15 Ascomycota genera, were included in this study to determine the specificity of the markers developed for *G. abietina *(Table 2). These fungi were selected taking into account their 18S rDNA-based phylogenetic relationships to *G. abietina *[34], so both closely related and divergent groups were included. Pure cultures of these fungi were used for DNA isolation. Phylogenetic study has shown that species in the *Helotiales *and *Rhytismatales *are closely related to *Gremmeniella *[35] and many of them are plant pathogenies. We could not include them in the tests due to the lack of fungal material. However, 18S rDNA sequences of 40 species of *Helotiales *and *Rhytismatales *were downloaded from GenBank for sequence analysis.

Fourteen conifer species from three families (Pinaceae, Cupressaceae and Taxodiaceae) were selected to represent the potential range of hosts (Table 2). Since pines are most susceptible to this pathogen, eight pine species native to Asia, Europe and North America were selected, representing the two *Pinus *subgenera: *Pinus *and *Strobus*. Three other reported hosts of *G. abietina *were also included: *Picea*, *Abies *and *Larix*. Seeds of each species were germinated on sterilized Petri dish for 2 – 3 weeks and used for DNA isolation. Twigs from six infected trees of *P. contorta *were collected in the forest of northern Sweden. From these, both brown and green needles were collected for DNA isolation.

The fungal genomic DNAs were isolated from a pure culture of each strain following the procedure described by Wu et al [36]. The genomic DNAs of the conifer species were isolated using a DNeasy^® ^Plant Mini Kit (Qiagen, Germany). The pine needles were thoroughly homogenized, as follows. Two ceramic beads, 4 mm in diameter (Iuchi, Japan) and 350 mg of 0.5 mm zirconia-silica beads (Biospec Products, Inc., Bartlesville, OK, USA), were placed in a 2-ml microtube containing 100 mg pine needles. The tubes were placed in a Mini-Bead Beater (Biospec Products, Inc.) and homogenized for 2 min at the maximum speed. The rest of the isolation procedure followed that suggested by the manufacturer of the DNeasy Plant Mini Kit (Qiagen, Germany).

### Specific primers

**Figure 1:**
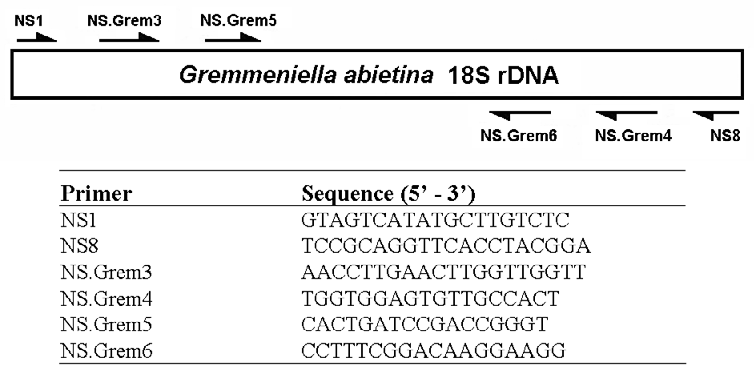
Location and sequences of the primers used in this study.

**Figure 2:**
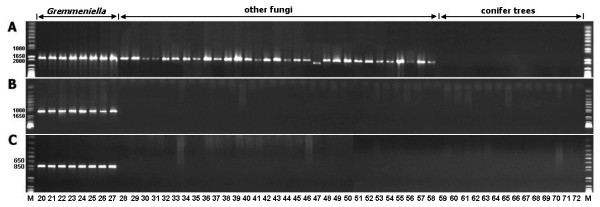
Specificity test of different PCR assays. A, amplification using fungal universal primer pair NS1/8; B and C, amplification using specific primer pairs NS.Grem3/4 and NS.Grem5/6, respectively. *G. abietina *isolates 1–19 were not shown on this gel due to space limitation. Nested PCR using NS.Grem3/4 and NS.Grem5/6 as inner primers gave amplification patterns identical to B and C, respectively. Fungal strains in each lane are in the order given in Table 1 and 2. M: 1 kb Plus DNA Ladder (Invitrogen).

**Figure 3:**
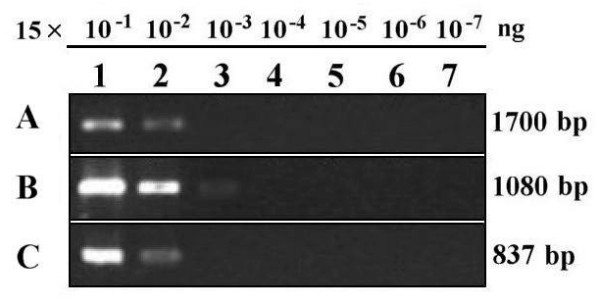
Comparison of detection sensitivity between single-step PCR assays. A, universal primers NS1/8; B, specific primers NS.Grem3/4; C, specific primers NS.Grem5/6. A 10-fold dilution series of *G. abietina *was used as the template.

**Figure 4:**
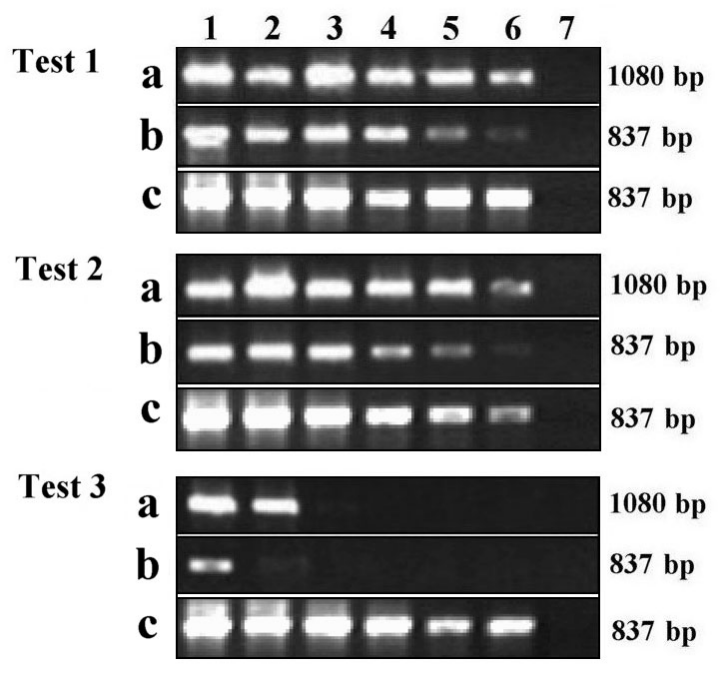
Comparison of the detection sensitivity between different nested PCR setups. Test 1, *G. abietina*; Test 2, *G. abietina *mixed with *P. contorta*; Test 3, *G. abietina *mixed with seven other fungi, in amplifications with NS1/8-NS.Grem3/4 (a), NS1/8-NS.Grem5/6 (b) and NS.Grem3/4-NS.Grem5/6 (c). See Table 3 for DNA contents in each lane.

**Figure 5:**
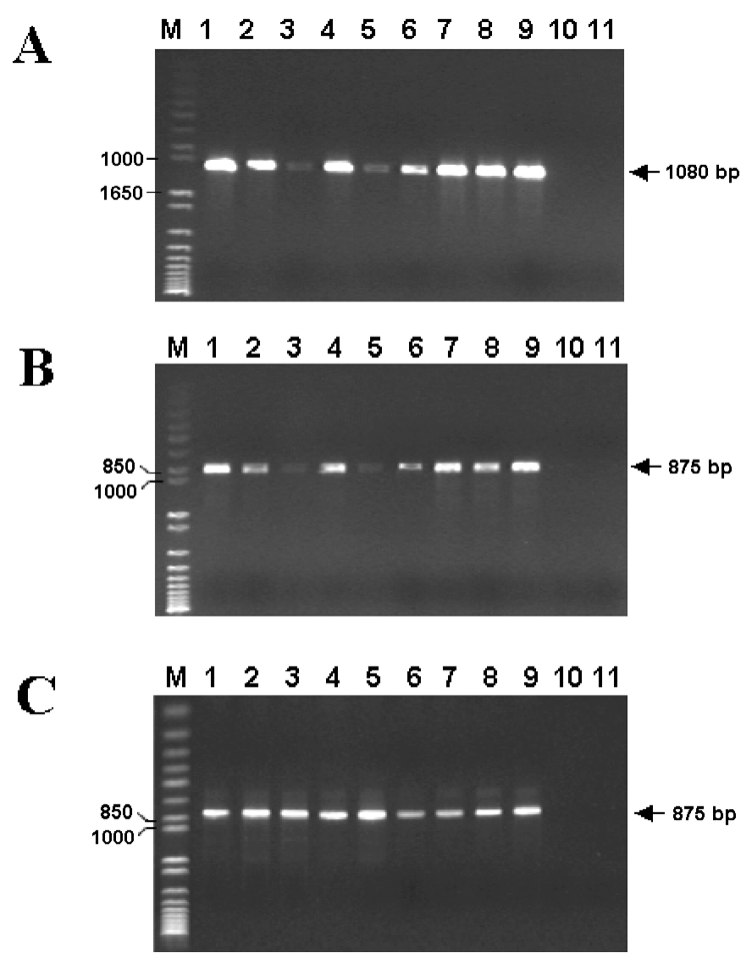
Detection of *G. abietina *in *P. contorta *trees. Lanes 1–6: needles from six infected twigs. Lanes 7–9: positive controls using *G. abietina *DNA. Lane 10: healthy *P. contorta*. Lane 11: negative control. M: 1 kb Plus DNA Ladder (Invitrogen). Panels A, B and C show the nested PCR results of NS1/8-NS.Grem3/4, NS1/8-NS.Grem5/6 and NS.Grem3/4-NS.Grem5/6, respectively.

The 11 *G. abietina *isolates sequenced for the 18S rRNA gene produced identical sequences, with one exception of a single substitution among them (GenBank accession numbers see Table 1). This sequence was aligned with the 18S rRNA sequences of the 31 other fungal strains listed in Table 2 as well as 40 *Helotiales *and *Rhytismatales *fungi accessed from GenBank (data not shown). Unique sequence patterns to *G. abietina *were utilized to design specific primers. Two pairs of primers, NS.Grem3/4 and NS.Grem5/6 (Fig. 1), were designed for *G. abietina*. To ensure the specificity of the PCR assay, these primers were first screened against sequences in GenBank using the BLAST function to examine their possible homology to other fungi. The "Search for short, nearly exact matches" program was used. These primers showed >20% mismatches to any other fungal sequences in GenBank including the 40 18S rDNA sequences of *Helotiales *and *Rhytismatales*. Under stringent PCR conditions, a >20% mismatch between the target molecule and the primer would not result in specific amplification.

### Nested PCR

To increase the detection sensitivity, two nested PCR systems were developed. One approach used the 18S rDNA-based universal fungal primers NS1 and NS8 [37] as outer primers in the first round PCR. Amplification was performed in a volume of 25 μl containing 1 – 5 ng of template DNA, 10 pmol of each primer, 0.75 U of Taq DNA polymerase (Invitrogen Life Technologies, USA), 200 μM of each dNTP (Amersham Pharmacia Biotech, USA), and 1.5 mM MgCl_2_. PCR conditions were optimized to comprise an initial denaturation of 3 min at 95°C, followed by 36 cycles of 94°C for 30 s, 45°C for 45 s and 72°C for 90 s, followed by a final extension of 10 min at 72°C. A 1 μl aliquot of the first round PCR product was used as the template in the second round, using the *G. abietina*-specific primers NS.Grem3/4 or NS.Grem5/6. The PCR conditions for these two specific primer pairs were similar to the NS1/8 amplification, except that the PCR cycles were decreased to 25 in the second round PCR and the annealing temperatures were 60°C and 56°C for NS.Grem3/4 and NS.Grem5/6, respectively. In another approach, *G. abietina*-specific primer pair NS.Grem3/4 was used in the first round PCR and NS.Grem5/6 in the second round. The PCR procedure is the same as that described above. A negative control was included in all PCR runs. PCR products (3 μl) were analyzed by electrophoresis on 1.4% agarose gels in 1× TAE buffer. The gels were stained with ethidium bromide and visualized under UV light using a Gel Doc 2000 fluorescent gel documentation system (Bio-Rad, USA).

### Sensitivity evaluation

To determine the detection limit of the nested PCR, three DNA dilution series were created and subjected to the PCR analysis (Table 3). First, a 10-fold dilution series of *G. abietina *genomic DNA was tested (Test 1). The initial DNA concentration of 5 ng/μl was quantified using a GeneQuant Pro RNA/DNA Calculator spectrophotometer (Amersham Biosciences, Sweden). To simulate the detection of infection in a host, this dilution series of *G. abietina *DNA was mixed with genomic DNA (4 ng/μl) of *P. contorta *in equal volume (Test 2). A 3 μl aliquot of this mixture at each dilution (equivalent to 1.5 μl of *G. abietina *DNA solution plus 1.5 μl of *P. contorta *genomic DNA) was used in each PCR (Test 2). A third test was conducted to simulate the detection of *G. abietina *in a mixed fungal background. For this, genomic DNAs of seven fungi *Aspergillus ochraceus*, *Penicillium brevicompactum*, *Trichoderma viride*, *Eurotium herbariorum*, *Fusarium culmorum*, *Ulocladium botrytis *and *Phacidium infestans *were mixed in equal quantities. The final concentration of this composite DNA was 6 ng/μl. The 10-fold dilution series of *G. abietina *DNA was mixed with this composite fungal DNA in equal volume for PCR analyses (Test 3). A 3 μl aliquot of this mixture at each dilution was used in each PCR (Test 3). To compare the sensitivity and specificity of single-step PCR and nested PCR, the 10-fold dilution series of *G. abietina *genomic DNA was also analyzed directly with the inner specific primers NS.Grem3/4 and NS.Grem5/6 in a single-step PCR. All the experiments conducted in this study were repeated 3 – 5 times.

## Authors' contributions

XRW and QYZ designed and conducted the experiments. PH, provided the Swedish *G. abietina *isolates, and collected and identified the infected *P. contorta *twigs. All authors read and approved the final manuscript.

## Figures and Tables

**Table 1:** *Gremmeniella abietina *isolates examined in this study.

	Isolate	Sequenced	Strain type	Pine host	Origin
1	*G. abietina *ALI 531		EU, LTT	*P. contorta*	Vindeln, Sweden
2	*G. abietina *ALI 532		EU, LTT	*P. contorta*	Vindeln, Sweden
3	*G. abietina *ALI 533		EU, LTT	*P. contorta*	Vindeln, Sweden
4	*G. abietina *ALI 534		EU, LTT	*P. contorta*	Vindeln, Sweden
5	*G. abietina *ALI 569	DQ084550	EU, STT	*P. sylvestris*	Arctic circle, Finland
6	*G. abietina *ALI 570	DQ084551	EU, STT	*P. sylvestris*	Finland
7	*G. abietina *ALI 571	DQ084552	EU, STT	*P. sylvestris*	Finland
8	*G. abietina *ALI 572	DQ084553	EU, LTT	*P. sylvestris*	Finland
9	*G. abietina *ALI 573	DQ084554	EU, LTT	*P. sylvestris*	Finland
10	*G. abietina *ALI 574	DQ084555	EU, LTT	*P. sylvestris*	Finland
11	*G. abietina *US 810105	DQ084556	EU	*P. resinosa*	USA
12	*G. abietina *US 790048		EU	*P. resinosa*	USA
13	*G. abietina *CF 910032	DQ084557	NA	*P. banksiana*	Canada
14	*G. abietina *ALI G90	AF548076	EU, STT	*P. contorta*	Åsele, Sweden
15	*G. abietina *ALI G148	AF548075	EU, STT	*P. contorta*	Åsele, Sweden
16	*G. abietina *ALI G139	AF548074	EU, STT	*P. contorta*	Åsele, Sweden
17	*G. abietina *ALI G3		EU, STT	*P. sylvestris*	Nattavaara, Sweden
18	*G. abietina *ALI G4		EU, STT	*P. sylvestris*	Nattavaara, Sweden
19	*G. abietina *ALI G6		EU, STT	*P. sylvestris*	Nattavaara, Sweden
20	*G. abietina *ALI G15		EU, STT	*P. sylvestris*	Nattavaara, Sweden
21	*G. abietina *ALI G16		EU, STT	*P. sylvestris*	Nattavaara, Sweden
22	*G. abietina *ALI G17		EU, STT	*P. sylvestris*	Nattavaara, Sweden
23	*G. abietina *ALI F113		EU, STT	*P. contorta*	Hede, Sweden
24	*G. abietina *ALI F114		EU, STT	*P. contorta*	Hede, Sweden
25	*G. abietina *ALI F116		EU, STT	*P. contorta*	Ramsele, Sweden
26	*G. abietina *ALI F129		EU, STT	*P. sylvestris*	Östersund, Sweden
27	*G. abietina *ALI F174		EU, STT	*P. sylvestris*	Östersund, Sweden

**Table 2:** Other fungal strains and conifer species included in this study.

	**Fungal strains**		**Conifer species**
28	*Aspergillus niger *UPSC 1769	59	*Pinus sylvestris*
29	*Aspergillus ochraceus *UPSC 1983	60	*Pinus contorta*
30	*Aspergillus flavus *UPSC 1768	61	*Pinus massoniana*
31	*Aspergillus penicilloides *ALI 231	62	*Pinus banksiana*
32	*Aspergillus versicolor *UPSC 2027	63	*Pinus jeffreyi*
33	*Aspergillus silvaticus *ALI 234	64	*Pinus strobus*
34	*Cladosporium cladosporioides *ALI 50	65	*Pinus koraiensis*
35	*Chrysonilia sitophila *ALI 346	66	*Pinus yunnanensis*
36	*Eurotium herbariorum *ALI 216	67	*Picea abies*
37	*Fusarium culmorum *UPSC 1981	68	*Larix deciduas*
38	*Microdochium nivale *UPSC 3273	69	*Abies procera*
39	*Mucor plumbeus *UPSC 1492	70	*Chamaecyparis nootkatensis*
40	*Paecilomyces variotii *UPSC 1651	71	*Tsuga canadensis*
41	*Penicillium commune *CBS 343.51	72	*Taxodium distichum*
42	*Penicillium italicum *UPSC 1577		
43	*Penicillium chrysogenum *UPSC 2020		
44	*Penicillium brevicompactum *ALI 319		
45	*Penicillium frequentans *ALI 218		
46	*Rhizopus microsporus *UPSC 1758		
47	*Stachybotrys bisbyi *CBS 317.72		
48	*Stachybotrys chartarum *CBS 330.37		
49	*Stachybotrys dichroa *CBS 182.80		
50	*Stachybotrys oenanthes *CBS 252.76		
51	*Stachybotrys kampalensis *CBS 388.73		
52	*Stachybotrys microspora *CBS 186.79		
53	*Trichoderma reesei *QM 9414		
54	*Trichoderma viride *ALI 210		
55	*Ulocladium botrytis *CBS 173.82		
56	*Wallemia sebi *UPSC 2502		
57	*Phacidium infestans *A387		
58	*Phacidium infestans *A391		

**Table 3:** DNA dilutions and mixtures used in the sensitivity test. The relative abundance of *G. abietina *DNA to the other genomic background DNA is indicated in parentheses.

	**Test 1 ***G. abietina*	**Test 2 ***G. abietina *+ *P. contorta*	**Test 3 ***G. abietina *+ 7 fungi mix
Sample			
1	5 × 10^-1 ^ng/μl	5 × 10^-1 ^ng/μl + 4 ng/μl (1:8)	5 × 10^-1 ^ng/μl + 6 ng/μl (1:12)
2	5 × 10^-2 ^ng/μl	5 × 10^-2 ^ng/μl + 4 ng/μl (1:80)	5 × 10^-2 ^ng/μl + 6 ng/μl (1:120)
3	5 × 10^-3 ^ng/μl	5 × 10^-3 ^ng/μl + 4 ng/μl (1:800)	5 × 10^-3 ^ng/μl + 6 ng/μl (1:1200)
4	5 × 10^-4 ^ng/μl	5 × 10^-4 ^ng/μl + 4 ng/μl (1:8000)	5 × 10^-4 ^ng/μl + 6 ng/μl (1:12000)
5	5 × 10^-5 ^ng/μl	5 × 10^-5 ^ng/μl + 4 ng/μl (1:80000)	5 × 10^-5 ^ng/μl + 6 ng/μl (1:120000)
6	5 × 10^-6 ^ng/μl	5 × 10^-6 ^ng/μl + 4 ng/μl (1:800000)	5 × 10^-6 ^ng/μl + 6 ng/μl (1:1200000)
7	5 × 10^-7 ^ng/μl	5 × 10^-7 ^ng/μl + 4 ng/μl (1:8000000)	5 × 10^-7 ^ng/μl + 6 ng/μl (1:12000000)
	3 μl in PCR	1:1 vol. mix, 3 μl in PCR	1:1 vol. mix, 3 μl in PCR

## References

[B1] Donaubauer E (1972). Distribution and hosts of Scleroderris lagerbergii in Europe and North America. Eur J For Pathol.

[B2] Hellgren M (1995). Gremmeniella abietina - disease biology and genetic variation within Fennoscandia. Department of Forest Mycology and Pathology.

[B3] Skilling DD, Riemenschneider DE, Manion PD (1983). Screening conifers for resistance to Gremmeniella abietina.. Proc Int Symposium Scleroderris Canker of Conifers.

[B4] Barklund P, Rowe J (1981). Gremmeniella abietina (Scleroderris lagerbergii), a primary parasite in a Norway spruce dieback. Eur J For Path.

[B5] Hellgren M, Barklund P (1992). Studies of the life-cycle of Gremmeniella abietina on scots pine in southern Sweden. Eur J For Path.

[B6] Karlman M, Hansson P, Witzell J (1994). Scleroderris canker on lodgepole pine introduced in northern Sweden. Can J For Res.

[B7] Lang KJ, Schütt P (1974). Anatomische untersuchungen zur infektionsbiologie von Scleroderris lagerbergii Gr (Brunchorstia pinea (Karst.) von Höhn.). Eur J For Pathol.

[B8] Marosy M, Patton RF, Upper CD (1989). A Conducive Day Concept to Explain the Effect of Low-Temperature on the Development of Scleroderris Shoot Blight. Phytopathology.

[B9] Dorworth CE, Krywienczyk J (1975). Comparisons among isolates of Gremmeniella abietina by means of growth rate, conidia measurement, and immunogenic reaction. Can J Bot.

[B10] Uotila A (1983). Physiological and morphological variation among Finnish Gremmeniella abietina isolates. Commun Inst For Fenn.

[B11] Petrini O, Toti L, Petrini LE, Heiniger U (1990). Gremmeniella abietina and G. laricina in Europe: characterization and identification of isolates and laboratory strains by soluble protein electrophoresis. Can J Bot.

[B12] Hamelin RC, Ouellette GB, Bernier L (1993). Identification of Gremmeniella abietina races with random amplified polymorphic DNA markers. Appl Environ Microbiol.

[B13] Lecours N, Toti L, Sieber TN, Petrini O (1994). Pectic Enzyme Patterns as a Taxonomic Tool for the Characterization of Gremmeniella Spp Isolates. Can J Bot.

[B14] Müller MM, Uotila A (1997). The diversity of Gremmeniella abietina var. abietina FAST-profiles. Mycol Res.

[B15] Dusabenyagasani M, Lecours N, Hamelin RC (1998). Sequence-tagged sites (STS) for studies of molecular epidemiology of scleroderris canker of conifers. Theor Appl Genet.

[B16] McCartney HA, Foster SJ, Fraaije BA, Ward E (2003). Molecular diagnostics for fungal plant pathogens. Pest Manag Sci.

[B17] Grote D, Olmos A, Kofoet A, Tuset JJ, Bertolini E, Cambra M (2002). Specific and sensitive detection of Phytophthora nicotianae by simple and nested-PCR. Eur J Plant Path.

[B18] Renker C, Heinrichs J, Kaldorf M, Buscot F (2003). Combining nested PCR and restriction digest of the internal transcribed spacer region to characterize arbuscular mycorrhizal fungi on roots from the field. Mycorrhiza.

[B19] Hamelin RC, Bourassa M, Rail J, Dusabenyagasani M, Jacobi V, Laflamme G (2000). PCR detection of Gremmeniella abietina, the causal agent of Scleroderris canker of pine. Mycol Res.

[B20] O'Donnell K (1992). Ribosomal DNA internal transcribed spacers are highly divergent in the phytopathogenic ascomycete Fusarium sambucinum (Gibberella pulicaris). Curr Genet.

[B21] Gernandt DS, Liston A, Pinero D (2001). Variation in the nrDNA ITS of Pinus Subsection Cembroides: Implications for Molecular Systematic Studies of Pine Species Complexes. Mol Phylogenet Evol.

[B22] James TY, Moncalvo JM, Li S, Vilgalys R (2001). Polymorphism at the ribosomal DNA spacers and its relation to breeding structure of the widespread mushroom Schizophyllum commune. Genetics.

[B23] Ko KS, Jung HS (2002). Three nonorthologous ITS1 types are present in a polypore fungus Trichaptum abietinum. Mol Phylogenet Evol.

[B24] Dorworth CE (1972). Epidemiology of Scleroderris lagerbergii in central Ontario. Can J Bot.

[B25] Skilling DD, Schneider B, Dasking D (1986). Biology and control of scleroderris canker in North America.

[B26] Nevalainen S (1999). Gremmeniella abietina in Finnish Pinus sylvestris stands in 1986-1992: a study based on the National Forest Inventory. Scand J Forest Res.

[B27] Dorworth CE, Krywienczyk J, Skilling DD (1977). New York isolates of Gremmeniella abietina (Scleroderris lagerbergii) identical in immunogenic reaction to European isolates. Plant Dis Rep.

[B28] Hamelin RC, Lecours N, Laflamme G (1998). Molecular evidence of distinct introductions of the European race of Gremmeniella abietina into North America. Phytopathology.

[B29] Hansson P, Wang XR, Szmidt AE, Karlman M (1996). RAPD variation in Gremmeniella abietina attacking Pinus sylvestris and Pinus contorta in northern Sweden. Eur J For Path.

[B30] Hantula J, Muller MM (1997). Variation within Gremmeniella abietina in Finland and other countries as determined by Random Amplified Microsatellites (RAMS). Mycol Res.

[B31] Wang XR (1997). Genetic variability in the canker pathogen fungus, Gremmeniella abietina. contribution of sexual compared with asexual reproduction. Mycol Res.

[B32] Hansson P (1998). Susceptibility of different provenances of Pinus sylvestris, Pinus contorta and Picea abies to Gremmeniella abietina. Eur J For Path.

[B33] Kaitera J, Seitamaki L, Jalkanen R (2000). Morphological and ecological variation of Gremmeniella abietina var. abietina in Pinus sylvestris, Pinus contorta and Picea abies sapling stands in northern Finland and the Kola Peninsula. Scand J Forest Res.

[B34] Wu Z, Tsumura Y, Blomquist G, Wang XR (2003). 18S rRNA Gene Variation among Common Airborne Fungi, and Development of Specific Oligonucleotide Probes for the Detection of Fungal Isolates. Appl Environ Microbiol.

[B35] Gernandt DS, Platt JL, Stone JK, Spatafora JW, Holst-Jensen A, Hamelin RC, Kohn LM (2001). Phylogenetics of Helotiales and Rhytismatales based on partial small subunit nuclear ribosomal DNA sequences. Mycologia.

[B36] Wu Z, Wang XR, Blomquist G (2002). Evaluation of PCR primers and PCR conditions for specific detection of common airborne fungi. J Environ Monit.

[B37] White TJ, Bruns T, Lee S, Taylor J, Innis MA, Gelfand DH, Sninsky JJ and White TJ (1990). Amplification and direct sequencing of fungal ribosomal RNA genes for phylogenetics. PCR protocols: A guide to methods and applications.

